# Where Does Mediator Bind *In Vivo*?

**DOI:** 10.1371/journal.pone.0005029

**Published:** 2009-04-03

**Authors:** Xiaochun Fan, Kevin Struhl

**Affiliations:** Department of Biological Chemistry and Molecular Pharmacology, Harvard Medical School, Boston, Massachusetts, United States of America; University of Hong Kong, Hong Kong

## Abstract

**Background:**

The Mediator complex associates with RNA polymerase (Pol) II, and it is recruited to enhancer regions by activator proteins under appropriate environmental conditions. However, the issue of Mediator association in yeast cells is controversial. Under optimal growth conditions (YPD medium), we were unable to detect Mediator at essentially any *S. cerevisiae* promoter region, including those supporting very high levels of transcription. In contrast, whole genome microarray experiments in synthetic complete (SC) medium reported that Mediator associates with many genes at both promoter and coding regions.

**Principal Findings:**

As assayed by chromatin immunoprecipitation, we show that there are a small number of Mediator targets in SC medium that are not observed in YPD medium. However, most Mediator targets identified in the genome-wide analysis are false positives that arose for several interrelated reasons: the use of overly lenient cut-offs; artifactual differences in apparent IP efficiencies among different genomic regions in the untagged strain; low fold-enrichments making it difficult to distinguish true Mediator targets from false positives that occur in the absence of the tagged Mediator protein. Lastly, apparent Mediator association in highly active coding regions is due to a non-specific effect on accessibility due to the lack of nucleosomes, not to a specific association of Mediator.

**Conclusions:**

These results indicate that Mediator does not bind to numerous sites in the yeast genome, but rather selectively associates with a limited number of upstream promoter regions in an activator- and stress-specific manner.

## Introduction

The Mediator complex associates with RNA polymerase (Pol) II, and it is recruited to enhancer regions by activator proteins under appropriate environmental conditions [1]–[7]. Mediator stimulates basal Pol II transcription *in vitro*, and several subunits of Mediator are essential for general Pol II transcription in yeast cells [8], [9]. These and other observations have led to the view that Mediator is a general and essential component of the Pol II machinery *in vivo* that is central to the transduction of activation signals from enhancer-bound activators to general transcription factors.

We challenged this view by showing that Mediator does not detectably associate with many highly active Pol II promoters in *S. cerevisiae* cells grown under optimal conditions [10]. In fact, whole-genome microarray experiments performed under such conditions (YPD medium) yielded few, if any, specific Mediator targets. Furthermore, in response to heat shock and other stress conditions, Mediator is recruited to enhancer regions, but its association is not directly related to the level of Pol II association and in some cases is not detectable at highly activated promoters. Thus, we concluded that Mediator is recruited to enhancers in an activator-specific manner, and that it does not appear to be a general component of the active Pol II machinery *in vivo* [10].

In contrast to our results, other whole genome microarray experiments reported that Mediator associates with many genes at both promoter and coding regions in *S. cerevisiae* [11] and *S. pombe* [12] cells grown under non-stressed conditions (synthetic complete medium). Here, we investigate the apparent discrepancy between these studies. For *S. cerevisiae*, we show that part of this discrepancy is due to the growth medium used in the different studies, but that most Mediator targets identified in the genome-wide study [11] are false positives that arise for a number of interrelated reasons.

## Materials and Methods

### Yeast strains

All strains for ChIP experiments were derived from *S. cerevisiae* strain BY4742 and contain three copies of HA tag at the C-terminus of Med15. These strains were generated by insertion of a PCR fragments with a *URA3* selective marker to the C-terminus of Med15, followed by looping out this marker through homologous recombination [13]. Yeast cells were grown in YPD (1% yeast extract; 2% peptone; 2% glucose) or SC (1% yeast nitrogen base with ammonium sulfate; amino acids mixture; uracil; 2% glucose) medium to OD_600_ of about 0.6 to 0.8 before being fixed with 0.1% formaldehyde. Alternatively, cells were grown in YP medium supplied with 2% galactose or 1% galactose/1% glucose mixture. Heat shock experiments were performed by growing cells in YPD to early log phase, followed by 8 minutes heat shock at 39°C.

### Chromatin immunoprecipitation

Chromatin extracts preparation and chromatin immunoprecipitation were performed by standard methods [14] using antibodies to the HA epitope (F7 for Med15), TBP, Hsf1, or Gal4 DNA binding domain. Quantitative PCR were performed in real time using an Applied Biosystems 7000 Fast Real-time PCR System. For each genomic region, the IP efficiency was determined by comparing the amount of DNA in input and IP samples. The fold-enrichment of genomic regions was determined by normalizing IP efficiency to that of an ORF-free region from chromosome V unless otherwise stated. All values represent averages from three independent experiments and the error is ±25%.

### Data analysis

Microarray data from Andrau et al [11] were downloaded from http://www.molecule.org/cgi/content/full/22/2/179/DC1/. Ranks of targets were determined by sorting Mediator.BR descending for 1241 identified targets and the rest of non-targets separately. For some analyses, the binding ratio of every genomic region from non-tagged strain was subtracted from that of Mediator or Med3 to get the net binding. The number of targets for different Mediator subunits and the non-tagged strain were determined by counting corresponding binding ratios (BR) greater than the indicated cut-offs. Correlations between different Mediator subunits and non-tagged strain were calculated with Microsoft Excel for 1241 targets identified in Andrau et al [11].

## Results

### Differential Mediator association in YPD and SC media

In previous work, we showed that Mediator associates with enhancers in an activator-specific manner in *S. cerevisiae* [10]. In contrast, other studies in *S. cerevisiae* [11] and *S. pombe* [12] claimed that Mediator associates with numerous genomic regions in non-stressed conditions. This discrepancy is unlikely to involve differences in the Mediator subunits examined in the various studies, because each study examined occupancy of multiple Mediator subunits and subunit-specific differences were not observed.

**Figure 1:**
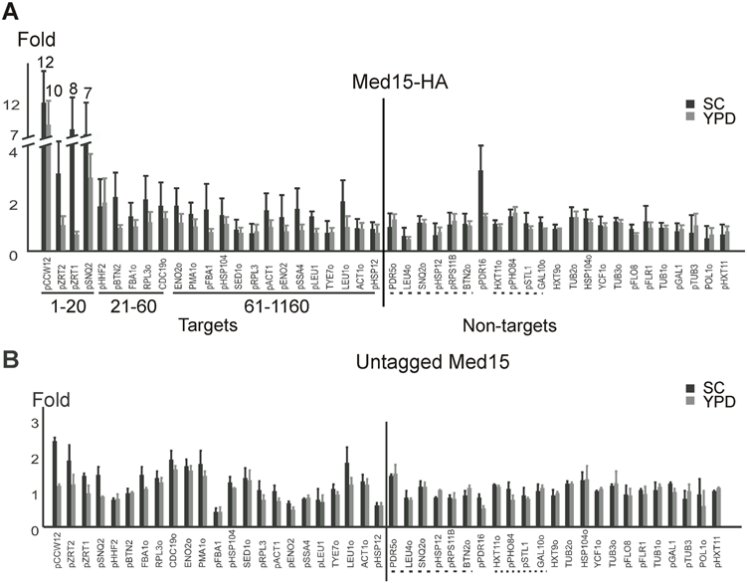
Mediator association at putative target and non-target regions in SC and YPD medium. (A) Association of Med15-(HA)_3_ was determined at the indicated genomic regions as described previously [10]. Relative occupancy values were calculated by determining the apparent immunoprecipitation efficiency (ratio of immunoprecipitated to input DNA) and normalized to the average value of 10 non-target regions (underlined with dashes), which was defined as 1 and served as the internal control. All values represent the mean of at least three independent experiments and the standard deviation is indicated. The indicated ranks of genomic regions are taken from Andrau et al. (2006) by sorting by Mediator.BR descending of the 1241 targets (gene names: p, promoter regions; o, ORF regions) and the rest of non-targets separately. (B) Control experiment with an isogenic yeast strain expressing untagged Med15. Relative occupancy values were normalized to the average of 10 non-target regions.

One possible explanation is that our studies [10] were performed primarily in rich broth (YPD), whereas those of Andrau et al [11] were performed in synthetic complete (SC) medium. We therefore used real-time, quantitative PCR analysis to analyze Med15 association with a large number of genomic regions with different levels of Mediator association as determined by Andrau et al [11] in both SC and YPD medium (Fig. 1A). The Med15 subunit was chosen, because it yields the highest fold-enrichments of 4 Mediator subunits we have examined previously [10] and hence represents the most sensitive assay for Mediator association. We used the average of 10 non-targets as a control value in order to minimize the bias and experimental error that would arise from using a single genomic region as the control. We define a target as having >2-fold enrichment above this control value; this commonly used definition corresponds to a *p* value of <0.05 for an experiment involving 3 biological replicates with a standard deviation that is ±25% of the mean [15].

In SC medium, Mediator association with the upstream region of *CCW12* is about 25 fold higher than with the coding region of *POL1* gene, comparable to that observed by Andrau et al [11]. Interestingly, in all cases where Mediator association is detected in SC medium, the level of Mediator association is consistently lower in YPD medium, both in terms of immunoprecipitation (IP) efficiency and fold-enrichment over background (Fig. 1A and data not shown). With two exceptions (*CCW12* upstream region and to a lesser extent *SNQ2*), Mediator association in YPD medium was not significantly above background levels (see below), consistent with our previous analysis [10]. Part of the *CCW12* upstream region was not present on the microarray used in our earlier analysis, indicating that there is at least one, and possibly a few more, genomic regions bound by Mediator in YPD medium. Thus, differences in growth medium account for part of the apparent discrepancy between the two studies. Although yeast cells grow rapidly in both SC and YPD medium, SC is less optimal than YPD, consistent with our previous suggestion that Mediator is recruited to genomic regions under non-optimal growth conditions. Presumably, some transcriptional activator proteins are functional in SC, but not YPD medium, thereby explaining differential Mediator recruitment.

### Quantitative PCR analysis suggests only 50–100 Mediator targets in SC medium

**Figure 2:**
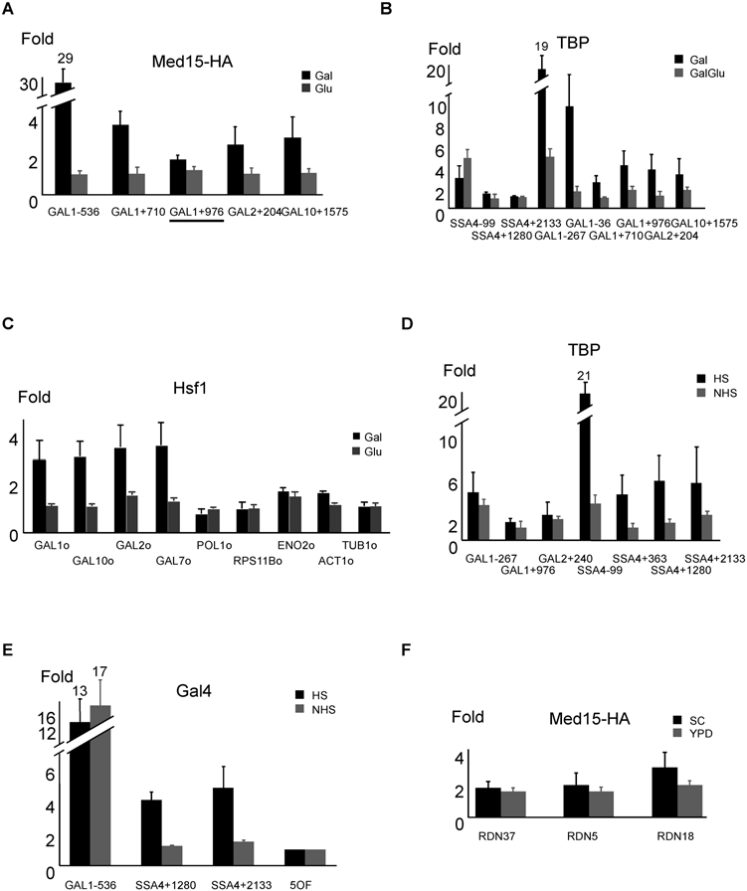
Highly transcribed regions give higher signals in ChIP experiments. (A) Mediator association at the indicated *GAL* regions in cells grown in galactose (gal) or glucose (glu). Primer pair *GAL1*+976 (underlined) is identical to that used by Zhu et al. (2006). Values were normalized to an ORF-free region on chromosome V, which was defined as 1. (B) TBP and (C) Hsf1 association at the indicated *GAL* regions in cells grown in galactose (Gal) or a mixture of glucose and galactose (GalGlu). (D) TBP and (E) Gal4 association with the indicated *GAL* and *SSA4* regions in cells grown in absence (NHS) or presence of an 8 min heat shock (HS) at 39°C. (F) Mediator association with genes highly transcribed by Pol I (*RDN18* and *RDN37*) and Pol III (*RDN5*).

Although Andrau et al [11] identified ∼1,200 Mediator targets in SC medium, we observed >2-fold enrichment for a much smaller number of genomic regions. Specifically, 4 out of 4 of the top 20 targets, 2 out of 5 targets ranked between 21 and 60 and 0 out of 14 targets ranked between 61 and 1160 showed >2-fold enrichment (Fig. 1A). Even when Mediator association is defined at this low level fold-enrichment, this analysis suggests that there are only 50–100 Mediator targets in SC medium. Only 3 out of 4 of the top 20 targets, and none of lower-ranked targets passed a 4-fold cutoff. In contrast, Mediator association at Gal4-, Hsf1-, and Ace1-dependent enhancers ranges between 20–50 fold over control regions ([10], [16]; Fig. 2A). It is unlikely that our failure to detect putative Mediator targets is due to non-optimal positioning of primer pairs, because the average size of sonicated chromatin ensures that Mediator binding profiles have relatively broad peaks, as evident at *GAL1*,*10* [10], [16] and *CCW12* [11].

Andrau et al [11] identified Mediator target sites by the standard approach of comparing relative IP efficiencies (IP: input ratios) among the large number of genomic regions on the microarrays. However, this approach also identifies “false positive” genomic regions that preferentially appear in the IP sample due to artifactual association with the agarose beads and/or the antibody (or something in the antibody preparation). Such artifactual interactions can give rise to reproducible 2-fold enrichments of some genomic regions over others [17], and hence can be observed in independent experiments involving strains in which individual proteins of a multiprotein complex are tagged with the same epitope. Furthermore, distinguishing between false positives and *bona fide* targets is difficult when fold-enrichments are in the 2–3 fold range, as is the case here for nearly all genomic regions. Indeed, in a parallel control experiment with an untagged strain (Fig. 1B), many genomic regions show ∼2-fold enrichment above the signal of the region giving the lowest signal.

### Increased non-specific association of proteins with highly transcribed regions

It has been claimed that Mediator associates with many coding regions in *S. cerevisiae* [11] and in *S. pombe* [12]. In *S. cerevisiae*, Mediator association with coding regions was reported to be strongly biased to highly transcribed genes [11] and observed at *GAL1* specifically under inducing conditions [12]. We could only confirm 1 out of 3 coding regions among the top 60 targets and none out of 6 coding regions ranked between 121 and 980 by real-time quantitative PCR analysis (*RPL3*, Fig. 1A). We confirmed weak Mediator association (3-fold enrichment above control genomic loci) throughout the *GAL1* coding region under inducing conditions (Fig. 2A), but did not observe the peak 1 kb downstream from the initiation site described previously [12]. This low level of Mediator association is far below the 20–30 fold enrichment observed at the *GAL* enhancer, consistent with previous studies [10], [16], [18]. Importantly, we observed a similar 3-fold increase in TBP and Hsf1 occupancies within the *GAL* coding region upon galactose induction (Fig. 2B, C), and elevated TBP and Gal4 occupancy at the coding regions of a heat shock gene (*SSA4*) upon heat shock (Fig. 2D, E).

These observations strongly suggest that, in comparison to typical genomic regions, highly transcribed coding regions are non-specifically more accessible to nuclear proteins *in vivo* and to antibody preparations used in the IP experiment. We presume that this non-specific accessibility of DNA in highly transcribed coding regions is due to the nucleosome depletion that occurs under conditions of high rates of Pol II elongation [19]–[21]. In accord with this suggestion, we also observed 2–3 fold enrichment of Mediator at genes highly transcribed by RNA polymerases I and III (Figure 2F). Taken together, these results suggest that the apparent association of Mediator with highly transcribed coding regions reflects a non-specific increase in accessibility of nucleosome-depleted DNA to nuclear proteins and not a specific interaction of Mediator.

### Reanalysis of microarray data for Mediator association

Andrau et al [11] identified Mediator targets on the basis of having a binding ratio >1.2, a very non-stringent cut-off. Indeed, our re-analysis of their published data indicates that even more “targets” are found when this same cut-off is applied to the control experiment involving an untagged strain, and this is true even when more stringent cut-offs are used (Table 1). In addition, the correlation coefficient for Mediator targets identified by different subunits was only modest (average 0.33; Table 2), and it is unclear how much of this weak correlation is due to Mediator or to the epitope tag used for all the subunits. This correlation coefficient is far below that typically observed for biological replicates (0.8 to 0.9) or for subunits of a common complex [22]–[25]. These observations strongly suggest that most Mediator targets identified by Andrau et al [11] are indistinguishable from false-positives identified in the control experiment, and hence are not *bona fide* targets of Mediator.

**Table 1:** Numbers of Mediator targets determined by different cut-offs.

Cut-off	Number of targets				
	Mediator	No TAP	Med3	Mediator- no TAP	Med3- No TAP
3	12	39	33	6	13
2	60	311	119	12	33
1.5	304	1395	411	20	57
1.2	1379	3729	1438	49	89

Numbers of targets determined by different cut-offs for Mediator (combining all subunits), Med3 and untagged strain from supplementary data of Andrau et al. (2006).In the last two columns, binding ratios from the untagged strain were subtracted from those of Mediator or Med3 respectively.

**Table 2:** Correlations between different subunits of Mediator.

	No TAP	Med15	Med3	Med7	Med14	Med19	Med17	CycC	AVG
No TAP	1	0.15	0.07	−0.1	0.10	0.06	−0.06	0.07	0.04
Med15		1	0.82	0.46	0.47	0.25	0.36	0.04	0.40
Med3			1	0.44	0.60	0.49	0.48	0.16	0.50
Med7				1	0.25	0.12	0.27	0	0.26
Med14					1	0.28	0.28	0.12	0.33
Med19						1	0.48	0.38	0.33
Med17							1	0.25	0.35
CycC								1	0.16

The correlation coefficiencies were calculated with Microsoft Excel for the 1241 targets identified in Andrau et al. (2006) between different Mediator subunits pairwise. The averages of correlation coefficiencies of a given subunit with all other subunits were listed in the last column.

## Discussion

We believe the controversy concerning Mediator targets in *S. cerevisiae* is resolved as follows. First, there are a small number of Mediator targets in SC medium that are not observed in YPD medium. As SC medium is a less favorable medium for growth than YPD, this observation is consistent with our previous suggestion that Mediator is selectively recruited to upstream regions in response to non-optimal conditions [10]. Second, most Mediator targets identified by Andrau et al [11] are false positives that arose for several interrelated reasons: the use of overly lenient cut-offs; artifactual differences in apparent IP efficiencies among different genomic regions in the untagged strain; low fold-enrichments making it difficult to distinguish true Mediator targets from false positives that occur in the absence of a tagged Mediator protein. In this regard, “non-specific” binding or other biochemical activities do not function equally on all DNA sequences. For example, the quality of DNA sequence motifs for DNA-binding proteins will vary among non-target regions simply by chance, thereby leading to small reproducible differences in the IP efficiency. It has been suggested that such weak, but reproducible, binding events in the *Drosophila* embryo are biologically insignificant [26]. Third, apparent Mediator association in highly active coding regions is due to a non-specific affect on accessibility due to the lack of nucleosomes, not to a specific association of Mediator. This non-specific association of proteins to highly active coding regions raises a potential concern for studies of transcriptional elongation. However, fold-enrichments of Pol II elongation factors at active coding regions are typically much higher than observed for the non-specific association described here. For example, both the Spt16 and Pob3 subunits of FACT show ∼12-fold enrichment at highly active genes [27]. Our analysis here does not address the large number of Mediator targets reported in *S. pombe* [12], although we note that many of the issues discussed here were not adequately addressed and are potentially problematic.

The findings presented here confirm our original conclusion that, in *S. cerevisiae*, Mediator is selectively recruited to a limited number of upstream promoter regions in an activator- and stress-specific manner [10]. Superficially, this conclusion appears to be in conflict with the observation that several Mediator subunits are essential for Pol II transcription [8], [9]. Although other explanations are possible [10], we strongly favor the view that Mediator plays a general and direct role in Pol II transcription as inferred by genetic and biochemical studies, but that Mediator associates only transiently with core promoters *in vivo*. Such a transient interaction would not be detected by chromatin immunoprecipitation, but would have an essential functional role. In this regard, the observation that Pol II association at promoters is equivalent to that throughout the corresponding coding region suggest that, in yeast cells, preinitiation complexes in yeast are unstable and the transition to elongation is rapid [28]. An unstable preinitiation complex in *S. cerevisiae* is further supported by the unusually large open complex between the TATA element and initiation site [29] and the related fact that initiation occurs downstream of where the preinitiation complex is formed (as defined by the TATA element which stererochemically fixes the location of TBP and other general factors). Thus, we speculate that the general and essential function of Mediator *in vivo* occurs during the rapid transition between initiation and elongation.
